# Foreword - Kidney disease in disadvantaged populations: An unconquered challenge

**DOI:** 10.5414/CNP86S100

**Published:** 2016-11-23

**Authors:** Keith C. Norris, Kowdle S. Prabhakar, Lawrence Agodoa, Guillermo García-García

**Keywords:** kidney disease, disadvantaged populations

## Abstract

No abstract available.

Disadvantaged communities in both emerging and developed countries, including ethnic minority groups, suffer disproportionately from kidney disease and its complications [1]. In many low and middle (low-mid) income countries, the burgeoning incidence and prevalence of chronic kidney disease (CKD) has overwhelmed the support for proper follow-up and management to retard its progression. Patients in these low-mid income countries are further disadvantaged by very limited access to general healthcare, restricted access to recommended prevention and early interventions at various stages of CKD, and limited or nonexistent end-stage renal disease (ESRD) support [2]. Support for ESRD care continues to be mostly economically driven, often restrained by limited trained or qualified personnel. Further, many patients continue to be exposed to an excess of environmental toxins and pollutants, which account for clustered cases of regional nephropathies [3, 4]. 

To address these concerns, the *10*^th^* International Conference on Kidney Disease in Disadvantaged Populations* was held on March 17^th^ and 18^th^, 2015, at the Old Mutual Conference and Exhibition Centre at Kirstenbosch, Cape Town, South Africa, as a satellite meeting of the International Society of Nephrology (ISN)/World Congress of Nephrology (WCN). The theme of the conference was “Challenges of Practice in a Resource Limited Environment: Focus on Africa”, addressing a critical region in the CKD landscape [5]. It was organized by the Committee for Kidney Health in Disadvantaged Populations, an advisory committee of the International Society of Nephrology (ISN CKHDP). The primary mission of the committee is to explore ways to reduce the burden of kidney disease in disadvantaged communities globally with special emphasis on low-mid income countries, and also in ethnic minority groups in developed countries that suffer disproportionately from kidney disease and its complications. This online supplement summarizes the presentations that were made at the meeting. 

Nearly 200 people attended the conference with representatives and presenters from multiple regions globally, including North, Central, and South America, Australia, Europe, Asia, and Africa. Conference activities were directed to issues facing Africa. Key topics included: 1) global burden of disease – prevalence and incidence of end-stage renal disease in selected regions and populations, 2) unique issues in African nephrology, 3) the role of kidney foundations in promoting kidney care in resource-limited environments, 4) nephrology in Africa – challenges of practice in a resource limited environment, and 5) developing kidney transplant programs in special populations. 

To address these key areas, stimulating presentations included novel early detection and prevention programs from several countries with a focus on kidney disease in Africa. A review of susceptibility factors contributing to progression of CKD, genetic and environmental factors accounting for unique regional nephropathies, availability and affordability of renal replacement therapy for the unfortunate patients who reach ESRD, and issues regarding organ allocation for the lucky few who have access to transplantation are detailed in the manuscripts published in this supplement of Clinical Nephrology. Unfortunately, the early detection programs in most of these economically-challenged countries are either not available or very limited and fragmented. An inspiring keynote address entitled “AKI – an unacceptable death sentence globally” was given by ISN president, Dr. Giuseppe Remuzzi. 

Over 30 junior faculty received financial support to attend and participate in the meeting. Awards were given to best posters and best oral presentations. Another highlight of the symposium included a moderated panel discussion on research development for young investigators working in low-resource, global health settings. Drs. Shuchi Anand, Bernadette Thomas, and John Stanifer moderated the panel. Speakers included Drs. Guillermo García-García, Lawrence Agodoa, Karen Yeates, Vivekanand Jha, Wendy Hoy, and Roberto Pecoits-Filho. The panel highlighted the work and career of several senior investigators who have been successfully undertaking research in this environment and have shared their experience in setting up projects and securing funding. The discussion focused on research priorities and specific challenges as they relate to young investigators as well as priorities for future work and the methodological approaches most likely to be successful (http://www.theisn.org/about-isn/isn-blog/item/1801-post-wcn-2015-pane…ssion-on-challenges-for-young-investigators-in-low-resource-settings). 

In summary, on this 10^th^ anniversary of organizing satellite meetings, the ISN CKHDP has come a long way in highlighting the unique problems faced at least in some of the low-mid income countries as well as disadvantaged communities within the developed countries. These satellite conferences have seen growing interest among physicians, researchers, and policy experts as evidenced by the increasing number of delegate attendance and abstract submission. Through these biennial symposia over the last 20 years, the ISN CKHDP has fostered participation of nephrology care providers from low-mid income countries in the ISN nephrology training programs as well as the ISN Kidney Disease Data Center (ISN-KDDC). In fact, the ISN-KDDC recently reported a 14.3% prevalence of CKD in the general population and 36.1% in high-risk populations in over 75,000 persons in 12 low-mid income countries from six world regions including Bangladesh, Bolivia, Bosnia and Herzegovina, China, Egypt, Georgia, India, Iran, Moldova, Mongolia, Nepal, and Nigeria [6]. The ISN CKHDP has been playing a key role by raising awareness of CKD among the most vulnerable persons and nations, linking together people from the developed and the developing world who strive to care for the disadvantaged and exchange ideas for providing renal care in resource-limited geographical areas. We believe the proceedings of this symposium captured in this special online supplement will be both a resource and an inspiration to individuals with a passion for reducing the global burden and improving outcomes for individuals with CKD and CKD risk factors. 
